# Assessment of the Role of Gefitinib With Concurrent Chemoradiation in Locally Advanced Head and Neck Cancer

**DOI:** 10.7759/cureus.32532

**Published:** 2022-12-14

**Authors:** Raju Prajapati, Om P Singh, Hameeduzzafar Ghori, Jagannath Jatav, Abhinav Narwariya, Vineeta Yogi, Essam Elbadawy Ismail, Mohammad Habeebur Raheman Shaikh, Shajiya Sarwar Moosa, Yousuf Begum

**Affiliations:** 1 Department of Radiation Oncology, Shyam Shah Medical College and Sanjay Gandhi Memorial Hospital, Rewa, IND; 2 Department of Radiation Oncology, Mahaveer Institute of Medical Sciences and Research, Bhopal, IND; 3 Department of Radiation Oncology, Gandhi Medical College, Bhopal, IND; 4 Department of Pathology, Shyam Shah Medical College and Sanjay Gandhi Memorial Hospital, Rewa, IND; 5 Department of Medical Oncology, Apollo Hospitals, Delhi, IND; 6 Department of Anatomy, Imam Abdulrahman Bin Faisal University, Dammam, SAU; 7 Department of Physiology, Imam Abdulrahman Bin Faisal University, Dammam, SAU

**Keywords:** recist criteria, metastasis, recurrent, residual, egfr inhibitor, gefitinib, locally advanced head and neck cancer

## Abstract

Background: The treatment of locally advanced head and neck carcinoma has been a combination of chemotherapy and radiation. The higher incidences of recurrence and metastasis warrant the search for an alternative therapy for better patient outcomes. This study was designed to evaluate the effect of gefitinib in conjunction with concurrent chemoradiation in locally advanced stages III and IV head and neck cancer.

Methodology: The patients were equally divided into two groups: Group I received cisplatin 100 mg/m2 on the first, 22nd, and 43rd days together with the radiation, whereas Group II was given the same treatment as Group I together with oral doses of gefitinib 250 mg on a daily basis, starting two weeks prior to radiotherapy and continuing until the completion of it. The dose of radiotherapy was 2 Gray (Gy) per fraction given over a period of five days per week to a maximum of 70 Gy in locally higher grades of head and neck neoplasms. The evaluation was performed in accordance with the RECIST (Response Evaluation Criteria in Solid Tumors) criteria, which include stable disease (SD), progressing disease (PD), partial response (PR), and complete response (CR). Salvage chemotherapy, potential surgical intervention, or palliative care was presented to patients with remaining or recurring diseases. The grading of the patients for acute and chronic radiation morbidity was done according to the Radiation Therapy Oncology Group (RTOG) criteria for toxicity during radiation treatment and at each subsequent follow-up. Parameters such as site, nodal involvement, stage, tumor status, and Eastern Cooperative Oncology Group (ECOG) were recorded.

Results: On comparing the patient characteristics, no statistical significance was observed. The overall response was seen in 24 (80%) and 28 (83.33%) patients in Group I and Group II, respectively (p = 0.08). All patients in Group I and Group II reported xerostomia as an acute/chronic adverse event of chemotherapy. Similarly, mucositis, dysphagia, and diarrhea were observed in all the patients, and no statistical difference was observed. Seventeen (56.67%) patients in Group II had complaints of skin rashes, while four (13.33%) patients in Group I had similar complaints (p = 0.01).

Conclusion: The study concludes that encouraging results were observed in comparing overall response after the addition of oral gefitinib to the traditional treatment of locally advanced head and neck neoplasms.

## Introduction

Head and neck cancer is the sixth most common cancer, accounting for more than 5% of all cases globally. Indian men are most commonly affected by this malignancy, which accounts for 23% of all cancers. Head and neck cancer accounts for 6% of all cancers in women [1]. The use of tobacco, alcohol consumption, and worse socioeconomic conditions are the culprits behind the higher incidence of head and neck malignancy among males as compared to other cancers [2].

Traditionally, a combination of radiotherapy and chemotherapy (chemoradiation) has been used for the management of locally advanced head and neck cancers. More than half of the treated patients develop recurrence and one-fourth of them develop metastatic disease in a period of two years post-chemoradiation [3].

The epidermal growth factor receptor (EGFR), a transmembrane glycoprotein and member of the ErbB (erythroblastic leukemia viral oncogene homolog) family of receptors, contains higher grades of tyrosine kinase activity inside its intracellular domain [3]. Through a number of phosphorylation-dependent signaling cascades that descend to transcription factors in the nucleus, EGFR activation promotes the sequence of differentiation, proliferation, and survival of cancer cells [4]. On the surface of healthy cells, EGFR expression is barely detectable [5]. However, it has been linked to the emergence of a number of cancers and has been observed in more than 30% of human body tumors and may be up to 90% of head and neck squamous cell cancers. Gefitinib (ZD1839, Iressa^TM^; AstraZeneca Inc., Cambridge, United Kingdom) inhibits the signal transduction pathways and is orally administered; it works on the mechanism by inhibiting EGFR tyrosine kinase [6,7]. It has been demonstrated that it inhibits cell proliferation in a dose-dependent way in human head and neck cancer cell lines [8,9]. Drugs that are found to be effective in oropharyngeal tumors include gemcitabine, methotrexate, taxanes, vinorelbine, and bleomycin [10,11]. However, tumor response rates seldom go over 30-35%, and responses are typically short-lived; therefore, the prognosis in cases of metastatic or recurrent cancer is dismal, with a median survival of just four months and one-year survival rates below 30% [12,13]. The goal of this study was to assess how gefitinib and concurrent chemoradiation treatment affected patients with locally advanced head and neck cancer.

## Materials and methods

After approval from the Institutional Review Committee (Gandhi Medical College, Bhopal, with letter number “IRB-2017-02-009”), this study was done at the tertiary care institute. All locally advanced oro/hypopharyngeal squamous cell carcinoma patients (stages III and IV, M0), aged between 18 and 70 years, who gave consent for the study were included in the study. However, the patients with initial stages of carcinoma (Grade I or II) and altered hepatic or kidney function were excluded from the study.

All the patients were randomly divided using the chit-and-box method into two groups of 30 patients each. Group I patients received radiation with simultaneous cisplatin 100 mg/m^2^ on the first, 22nd, and 43rd days, and Group II patients received radiation with simultaneous cisplatin together with oral gefitinib 250 mg started two weeks prior to radiotherapy on a daily basis till completion of it. The dose of radiotherapy was 2 Gray (Gy) per fraction given over a period of five days per week to a maximum of 70 Gy.

A response evaluation was performed once chemoradiotherapy was finished and patients were then followed up with every one to three months thereafter. The lesions with suspicion of residual or recurring disease were confirmed by performing a needle or tissue biopsy. RECIST (Response Evaluation Criteria in Solid Tumors) criteria were used for the evaluation of response, which included complete response (CR), partial response (PR), stable disease (SD), and progressing disease (PD). The patients with remaining or recurring diseases were treated with salvage chemotherapy, potential surgical intervention, or palliative care.

Patients were graded for acute and chronic radiation morbidity criteria according to the Radiation Therapy Oncology Group (RTOG) for toxicity during radiation treatment and at each subsequent follow-up. Late toxicities were those that developed beyond six months, while acute toxicities were those that emerged during or up to six months after therapy.

Parameters such as site, nodal involvement, stage, tumor status, Eastern Cooperative Oncology Group (ECOG), and smoking were recorded. The compilation of data was done in Excel and IBM SPSS Statistics for Windows, Version 21.0 (Released 2012; IBM Corp; Armonk, New York, United States) was used for its analysis. The quantitative and qualitative data were analyzed using the Student's t-test and the chi-square test, respectively. p < 0.05 was considered statistically significant.

## Results

All patients were successfully enrolled and completed the study. The mean age of Group I and Group II patients was 52.14 ± 8.75 years and 55.39 ± 9.19 years, respectively (p = 0.52). Similarly, gender distribution and body mass index (BMI) were found to be statistically insignificant between the groups (Table 1). The ECOG-0 was observed in 20 (66.67%) and 18 (60%) patients in Groups I and II, whereas ECOG-1 was found in 10 (33.33%) and 12 (40%) patients in Groups I and II, respectively. Twenty-three (76.67%) patients in Group I and 24 (80%) patients in Group II had a history of smoking (p = 0.02). The oropharynx was the more commonly affected site and was observed in 16 (53.33%) and 17 (56.67%) patients in Groups I and II, respectively (p = 0.52). Stage III was seen in 11 (36.67%) and 12 (40%) patients of Groups I and II, while Stage IV was observed in 19 (63.33%) and 18 (60%) patients of Groups I and II, respectively (p = 0.21). Tumor status I, II, III, and IV were observed in 0 (0%), five (16.67%), 13 (43.33%), and 12 (40%) patients in Group I, while one (3.33%), six (20%), 11 (36.67%) and 12 (40%) patients were in Group II (p = 0.93). Twelve (40%) and 13 (43.33%) patients had an N2 level of nodal status (p = 0.91) (Table 1).

**Table 1:** Baseline characteristics of patients. ^#^Data presented as mean ± SD or number (percentage). *p < 0.05. BMI: body mass index; ECOG: Eastern Cooperative Oncology Group.

Parameters		Group I (30 patients)^#^	Group II (30 patients)^#^	p value
Age (years)		52.14 ± 8.75	55.39 ± 9.19	0.52
Male:female		16:14	13:17	0.83
BMI (kg/m^2^)		53.19 ± 5.71	51.72 ± 6.23	0.18
ECOG	0	20 (66.67)	18 (60)	0.15
	1	10 (33.33)	12 (40)	
Smoking	Yes	23 (76.67)	24 (80)	0.02*
	No	7 (23.33)	6 (20)	
Site	Oropharynx	16 (53.33)	17 (56.67)	0.52
	Hypopharynx	14 (46.67)	13 (43.33)	
Stage	III	11 (36.67)	12 (40)	0.21
	IV	19 (63.33)	18 (60)	
Tumor status	I	0 (0)	1 (3.33)	0.93
	II	5 (16.67)	6 (20)	
	III	13 (43.33)	11 (36.67)	
	IV	12 (40)	12 (40)	
Nodal status	N0	2 (6.67)	4 (13.33)	0.91
	N1	6 (20)	7 (23.33)	
	N2	12 (40)	13 (43.33)	
	N3	10 (33.33)	6 (20)	

Table 2 shows that overall response was seen in 24 (80%) and 28 (83.33%) patients in Group I and Group II, respectively (p = 0.08). However, CR was seen in 12 (40%) patients in both groups (p = 1.00), whereas PR was observed in 12 (40%) in Group I and 16 (53.33%) in Group II (0.07). Proliferative disease was observed in 25 (83.33%) patients in Group I and 24 (80%) patients in Group II (p = 0.81) (Table 2).

**Table 2:** Response to treatment. ^#^Data presented as number (percentage).

Parameters	Variables	Group I^#^	Group II^#^	p value
Response	Overall	24 (80)	28 (93.33)	0.08
	Complete	12 (40)	12 (40)	1.00
	Partial	12 (40)	16 (53.33)	0.07
Proliferative disease		25 (83.33)	24 (80)	0.81

Acute/chronic adverse events of chemotherapy reported were xerostomia in all the patients of Group I and Group II (Table 3). Similarly, mucositis, dysphagia, and diarrhea were observed in all the patients and no statistical difference was observed (Table 3). Seventeen (56.67%) patients in Group II had complaints of skin rashes, while four (13.33%) patients in Group I had similar complaints (p = 0.01). The higher incidences of skin rash in Group II were due to the addition of gefitinib drug (Table 3). 

**Table 3:** Acute/chronic adverse events of chemotherapy. ^#^Data presented as mean ± SD or number (percentage). *p < 0.05.

Adverse events	Group I^#^	Group II^#^	p value
Xerostomia	30 (100)	30 (100)	1.00
Mucositis	30 (100)	30 (100)	1.00
Skin rashes	4 (13.33)	17 (56.67)	0.01*
Dysphagia	30 (100)	30 (100)	1.00
Diarrhea	30 (100)	30 (100)	1.00

## Discussion

The objective of this study was to evaluate the effect of gefitinib in conjunction with concurrent chemoradiation in locally advanced head and neck cancer. Our results showed that Group I comprised 16 males and 14 females and Group II had 13 males and 17 females. In Groups I and II, respectively, the overall response rates, including both complete and partial, were 62% and 71.42%, respectively, and no statistically significant difference was observed (p = 0.605). Group I had a progression-free survival of 24 months, while Group II had a median of 35 months. In one group, the overall survival was 31 months, whereas in the other group it was 37 months. On comparing the toxicity/side effects of both groups, no statistical significance was observed. Saini et al. observed in their study that among 67 patients 32 received radiation and cisplatin (Group I) and 35 received radiation and cisplatin plus gefitinib (Group II) [14].

Our results showed ECOG-0 in 20 and 18 patients in Groups I and II, whereas ECOG-1 was found in 10 and 12 patients in Groups I and II, respectively. The oropharynx was affected in 16 and 17 patients in Groups I and II, respectively. Cohen et al. assessed the effectiveness and side effects of gefitinib 250 mg daily in patients with recurrent and/or metastatic squamous cell carcinoma of the head and neck (SCCHN) [15]. Before and after treatment, transforming growth factor-alpha and vascular endothelial growth factor levels in the serum were measured. One PR (1.4%) was seen in 70 individuals. Adverse medication reactions of Grade 3 occurred in four patients. Sixty-four percent of subjects had rashes of any grade. There was a correlation between the degree of cutaneous toxicity and disease control (PR + stable illness), progression-free survival, and overall survival.

Our results showed that the overall response was seen in 80% and 93.33% of patients in Group I and Group II, respectively. In Group I, 12 patients had CR and the remaining 12 patients had PR. Similarly, in Group II, 12 patients had CR, whereas 16 patients had PR. Proliferative disease was seen in 24 and 25 patients of Groups I and II, respectively. The results of the Meta-analysis of Chemotherapy in Head and Neck Cancer Study (MACHNC), which comprised data from over 11,000 individual patients in 63 randomized studies, showed that adding chemotherapy to radiotherapy for locally advanced cancer increased overall survival by 8% at five years [16]. However, in the present study, we added gefitinib as a concurrent medication with chemoradiation, and no significant added response was observed in terms of patient outcomes. Singh et al. conducted a study comparing the effectiveness of gefitinib with or without simultaneous chemoradiation in 86 patients with head and neck squamous cell carcinoma [17]. They divided the patients into two groups, in which the study group composed of 43 patients was given 250 mg of gefitinib per oral together with cisplatin 30 mg/m^2^ and radiotherapy every week, and the control group was given only cisplatin 30 mg/m^2^ with radiotherapy on a weekly basis. In the study group, they found full responses in 34 patients and PRs in four patients. Statistically, a significant difference was observed in the overall response (88.37%, p < 0.05). However, 27 and three patients had CR and PR, respectively, in the control group. The overall response was observed in 69.76% of the patients.

Acute/chronic adverse events of chemotherapy were reported as xerostomia, mucositis, skin rashes, dysphagia, and diarrhea. All the patients had findings of xerostomia, mucositis, dysphagia, and diarrhea at some period of treatment. However, most of the presentations were of a milder grade and successfully treated on an outpatient department (OPD) basis. Group II had higher incidence of skin rash probably due to the side effect of gefitinib itself. In patients with Stage III or IV SCCHN (oro/hypopharyngeal or laryngeal), Bonner et al. showed that when radiation was used with cetuximab it showed superior response as compared to radiation alone. Along with this, the local control and the overall survival were observed to be clinically and statistically significant [18].

The trial was limited in that it was unable to determine overall survival and progression-free survival with the addition of gefitinib. Moreover, the limited number of participants also compromises the projection of the study toward its conclusion. A more comprehensive investigation with statistical power might discover differences in survival.

## Conclusions

A combination of gefitinib and cisplatin is well tolerated concurrently with radiation but does not have an impressive effect on response rate. The addition of gefitinib to the standard treatment of locally advanced head and neck cancer does not give benefits in terms of response to the treatment, and the addition of toxicity.
